# Supplementary material for: Amylin deposition activates HIF1α and 6-phosphofructo-2-kinase/fructose-2, 6-biphosphatase 3 (PFKFB3) signaling in failing hearts of non-human primates
Source: Commun Biol. 2021 Feb 12;4:188. doi: 10.1038/s42003-021-01676-3 (PMC7881154; doi:10.1038/s42003-021-01676-3)
Supplement: Supplementary file 2 — Supplementary Information [file 42003_2021_1676_MOESM2_ESM.pdf]

Supplemental materials:
Amylin deposition activates HIF1α and 6-phosphofructo-2-kinase/fructose-2,6-biphosphatase 3 (PFKFB3) signaling in failing hearts of non-human primates

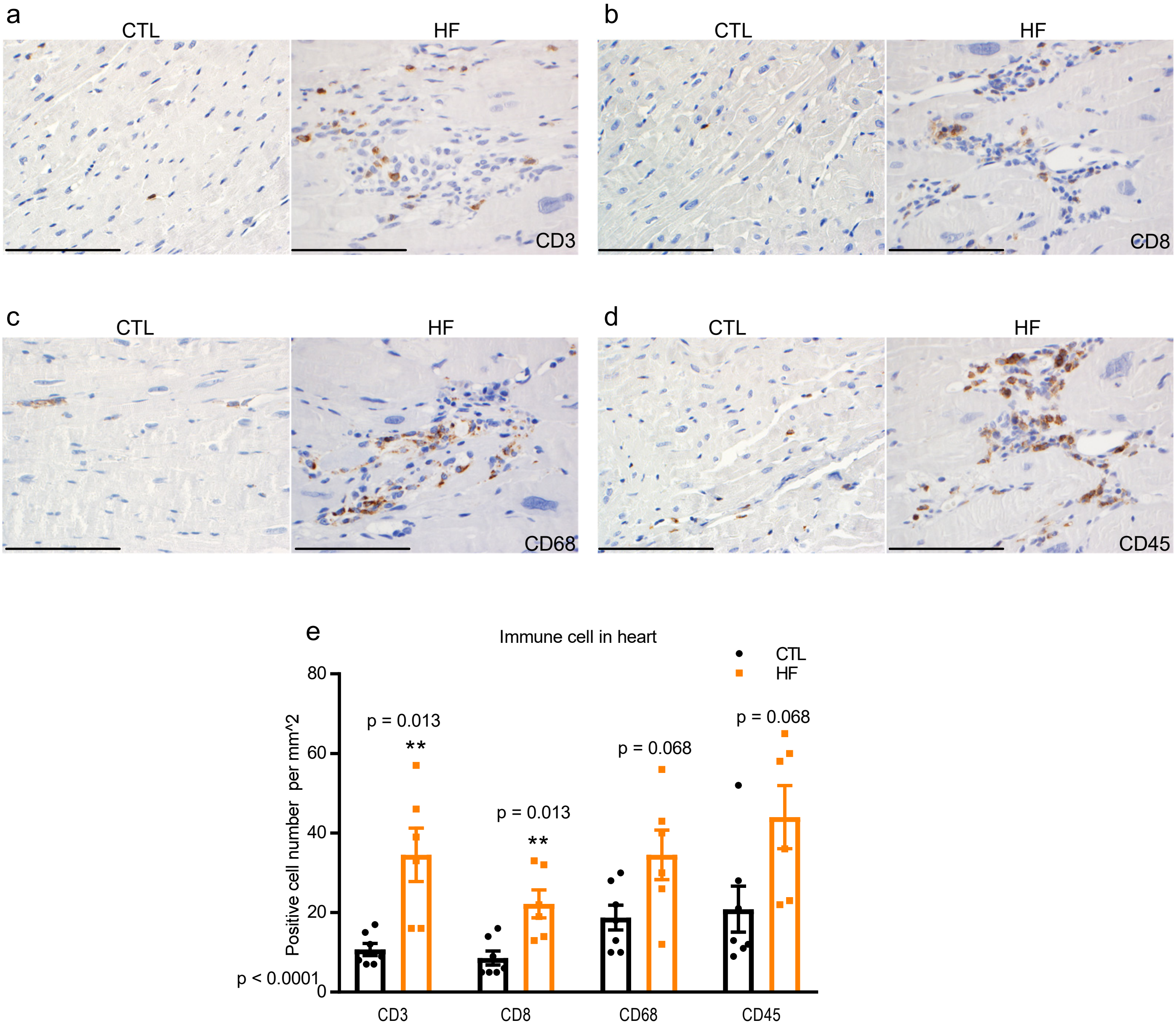

Supplementary Fig. 1. Expression of Immune cells in myocardium of NHPs. (a-d) Representative images of expression of CD3 (a) for T cells, CD8 (b) for cytotoxic T cells, CD68 (c) for macrophages, and CD45 (d) for leukocytes in hearts from NHPs with CTL (n = 7) and HF (n = 6). (e) Quantification of different expression levels of immune cells from NHPs with CTL (n = 7) and HF (n = 6) was shown in bar graph (e). Scale bar, 100 μm. Data represent mean ± SEM. **P<0.01 by Student's t-test.

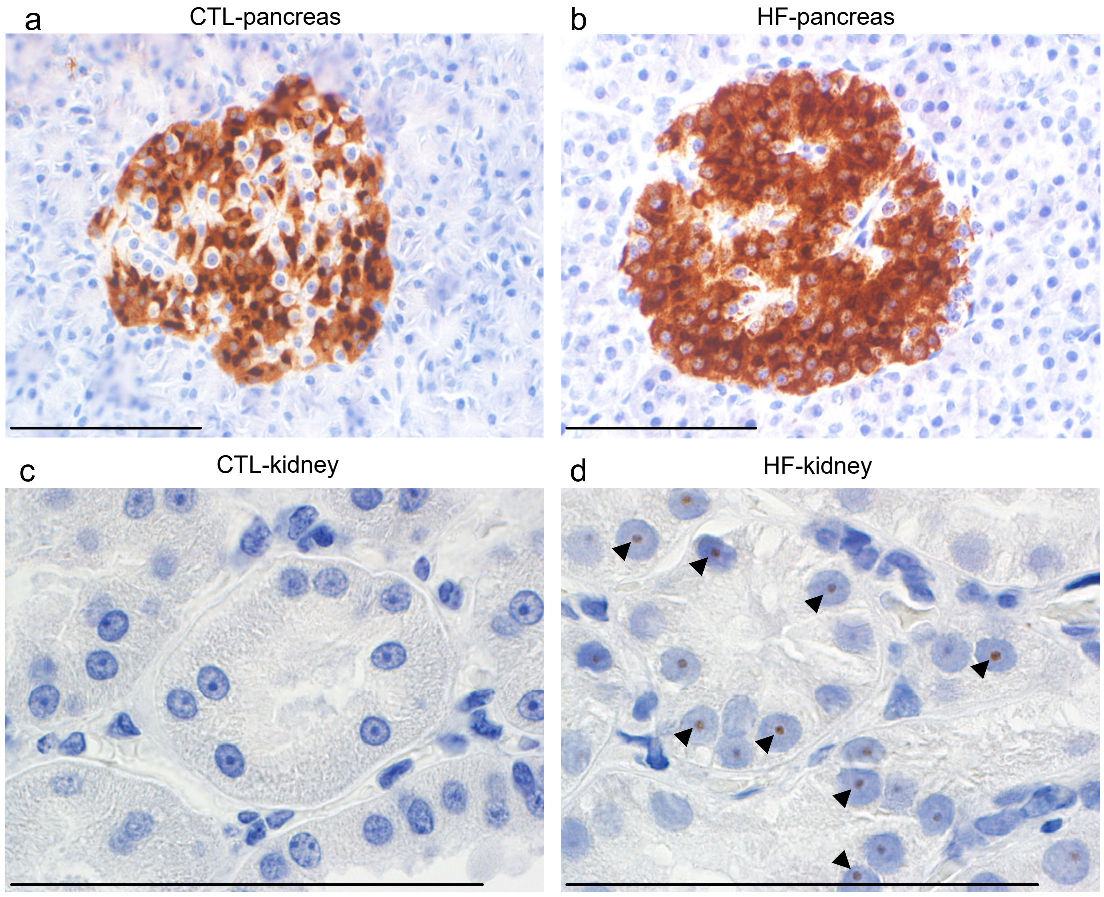

Supplementary Fig. 2. Amylin deposition (mouse anti-amylin antibody) in the pancreas (a-b) and kidneys (c-d) of NHPs with CTL and HF. Representative image for amylin deposition in the pancreas from CTL NHPs (n = 7) (a) and HF NHPs (n = 6) (b). No amylin deposits were observed in the CTL kidneys (c) (n = 7). Amylin deposition was shown in kidney nucleus (d, arrowhead) of the HF group (n = 6). Scale bar, 100 μm.

Supplementary Fig. 3

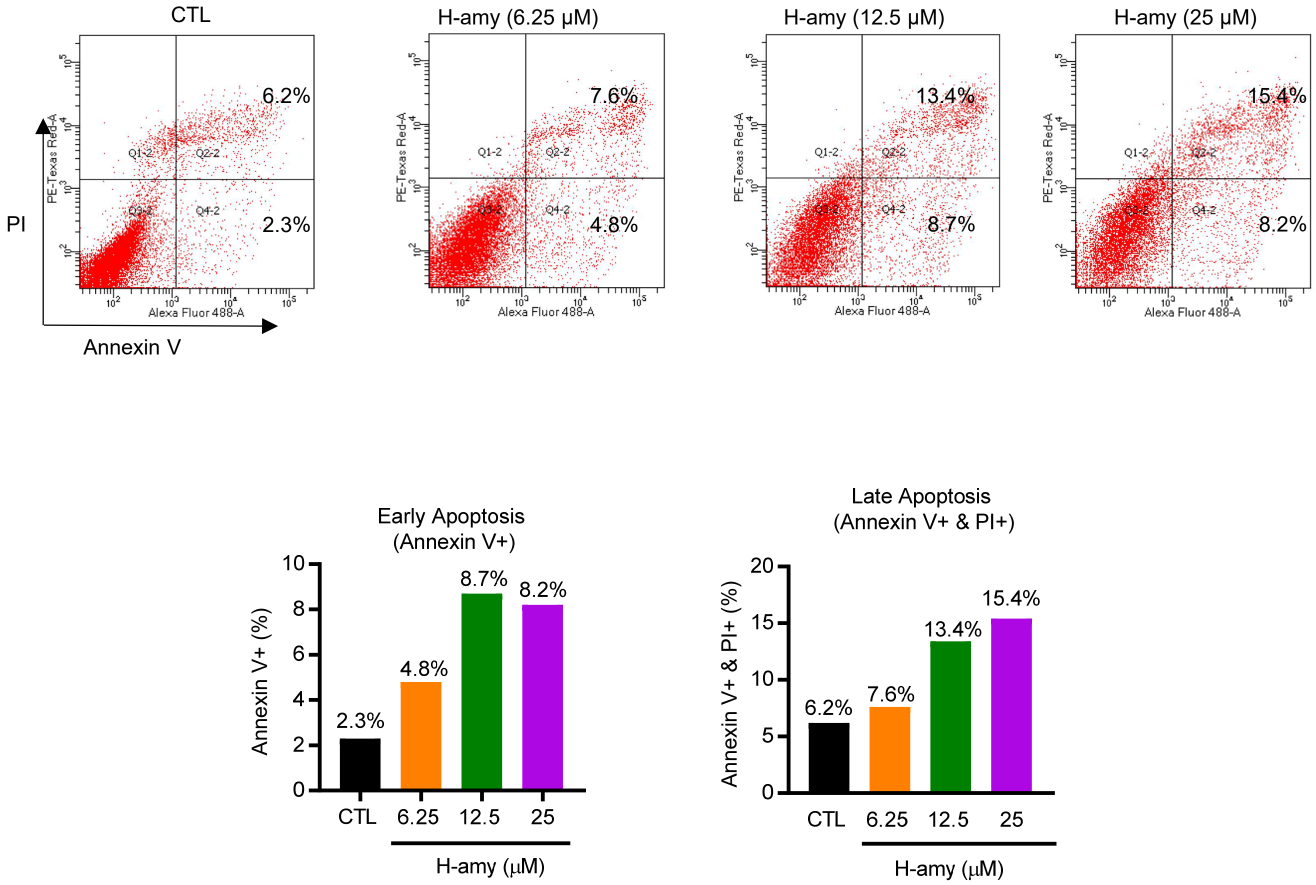

Supplementary Fig. 3. Human amylin-induced apoptosis in hiPSC-CMs was assessed by flow cytometry. hiPSC-CMs were incubated with preformed amylin oligomers at 6.25 μM, 12.5 μM, and 25 μM for 2 h at 37 °C, respectively. The percentages of cells with positive signals of Annexin V and/or PI were shown on top panels. The percentages of Annexin V+ (Early apoptosis) and Annexin V+& PI+ (Late Apoptosis) were compared between different doses of amylin treatment.

Supplementary Fig. 4

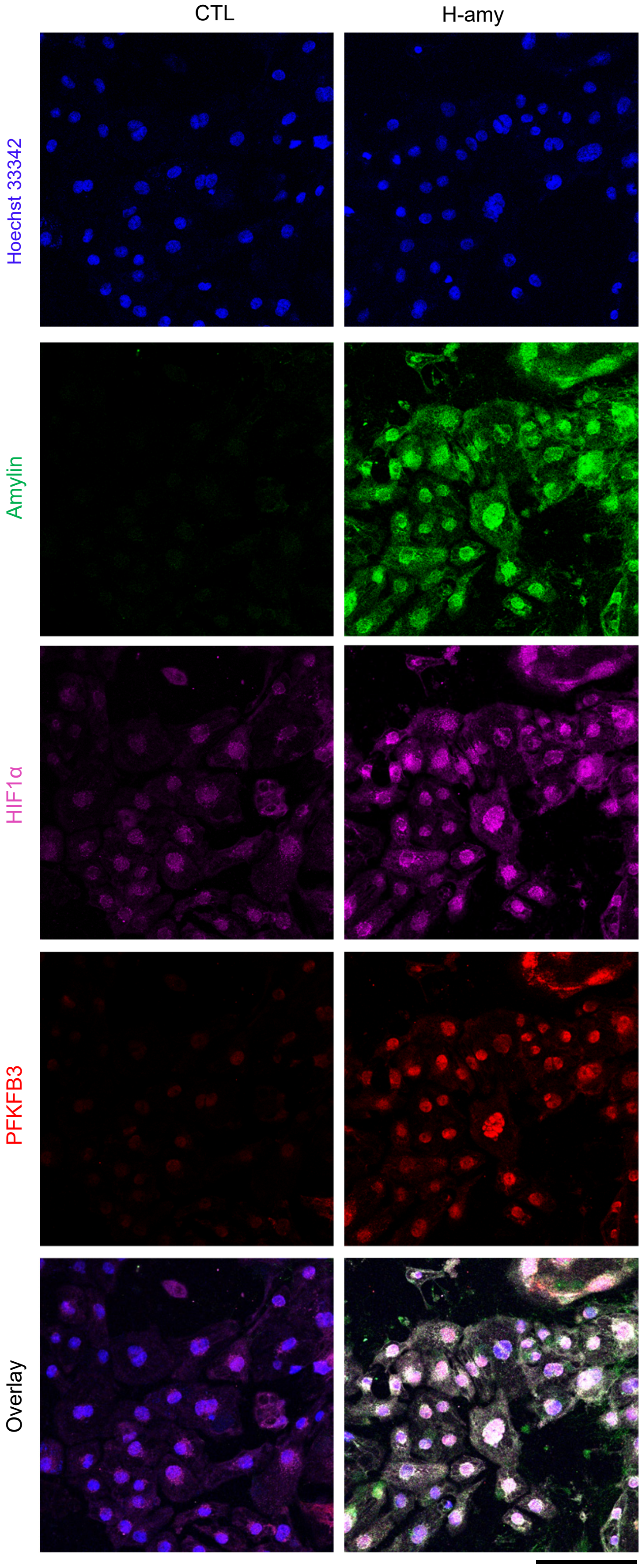

Supplementary Fig. 4. Cardiac amylin accumulation activated HIF1α and PFKFB3 in hiPSC-CMs. Co-Immunostaining of human amylin (green color, mouse anti-human amylin antibody), HIF1α (violet color, goat anti-HIF1α antibody), PFKFB3 (red color, rabbit anti-PFKFB3 antibody) and Hoechst 33342 (blue color) was shown in cells incubated with human amylin (H-amy) or without H-amy (CTL) (n = 5/group). Scale bar, 100 μm.

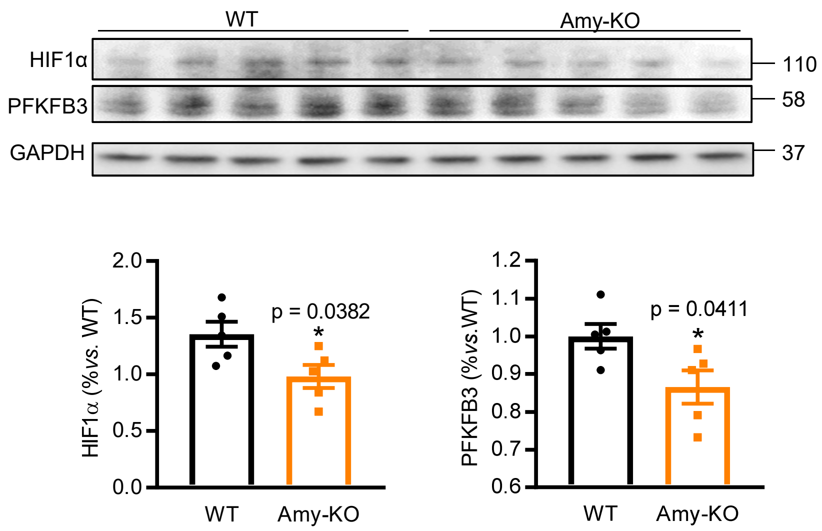

Supplementary Fig. 5. Decreased HIF1α and PFKFB3 levels in hearts from Amylin knock-out mice. Western blot analysis with anti-HIF1α and anti-PFKFB3 antibodies on heart homogenates from wild type (WT, n = 5) and amylin knock-out (Amy-KO, n = 5) mice. Data represent mean ± SEM. *P<0.05 by Student's t-test.

Supplementary Fig. 6

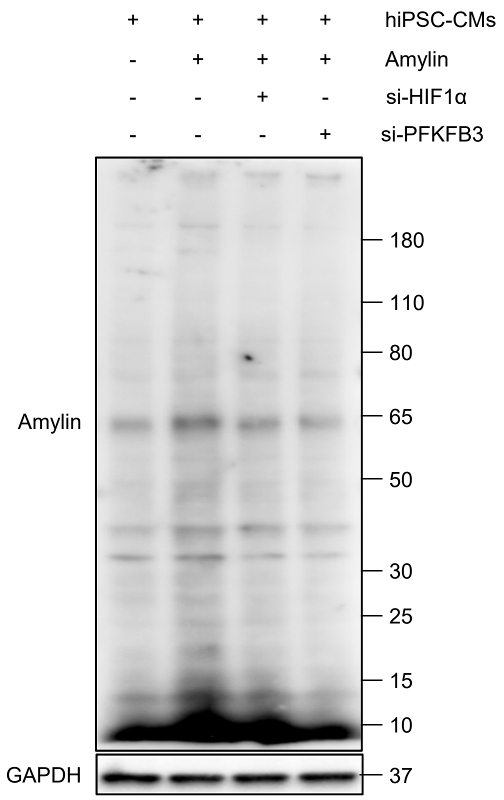

Supplementary Fig. 6. Decreased amylin deposition was assessed by western blot in hiPSC-CMs treated with siHIF1α and siPFKFB3, respectively, prior to amylin exposure.

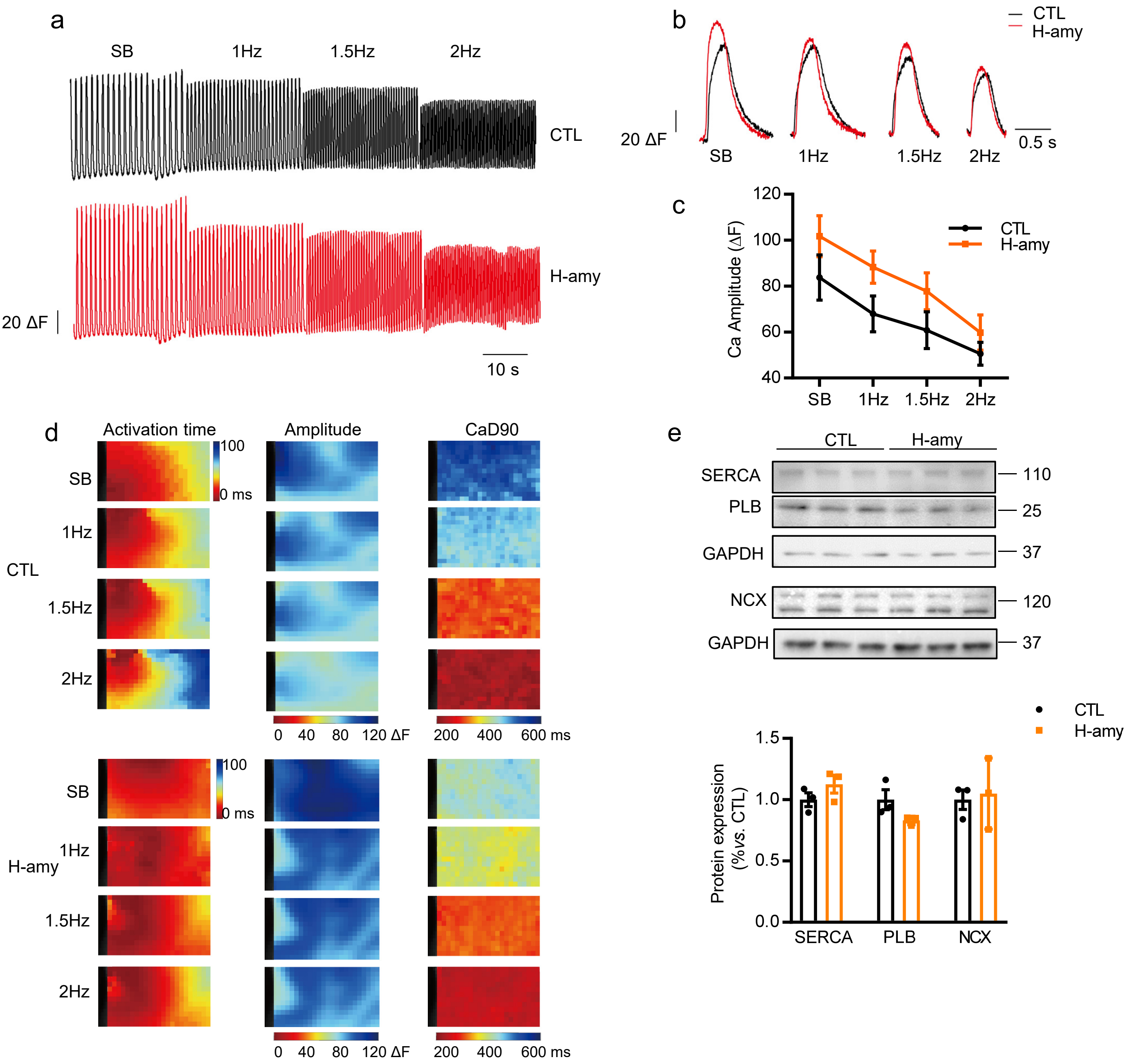

Supplementary Fig. 7. Calcium transients and Ca^{2+} cycling proteins in hiPSC-CMs treated with human amylin. (a) Representative Ca^{2+} transient measurements at spontaneous beating (SB) and electrically paced at 1Hz, 1.5Hz and 2Hz, respectively. (b) Representative superimposed Ca^{2+} transient traces in hiPSC-CMs treated without/with human amylin. (c) Mean amplitude of Ca^{2+} transients in hiPSC-CMs exposed to human amylin and CTL at spontaneous beating (SB) and electrically paced at 1Hz, 1.5Hz and 2Hz, respectively (n = 4/group). (d) Illustrative isochronal maps of calcium activation time, calcium amplitude and at different frequency. In the activation time map, warmer color means shorter activation time. In the amplitude map, blue color represents higher amplitude. In the CaD90 (the duration of calcium transient at 90% decay) map, red color means shorter duration. (e) No changes in the protein expression of sarcoplasmic reticulum Ca^{2+}-ATPase (SERCA), phospholamban (PLB) and Na^{+}/Ca^{2+} exchanger (NCX) in hiPSC-CMs treated with human amylin or CTL (n = 3/group). Data represent mean ± SEM.

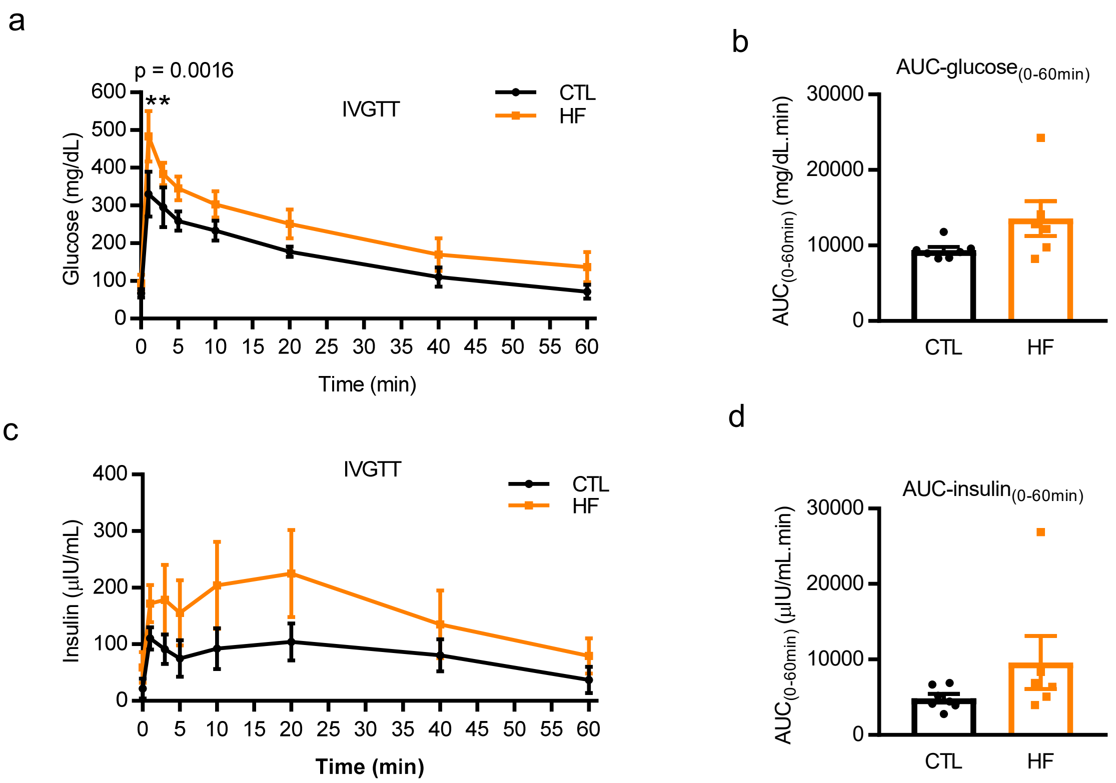

Supplementary Fig. 8. NHPs with HF showed impaired glucose tolerance. An *in vivo* intravenous glucose tolerance test (IVGTT), using a glucose dose of 0.5 g/kg body weight was performed in NHPs with CTL (n = 7) and HF (n = 6). (a) Plasma glucose concentration during IVGTT. (b) Area under curve for glucose during IVGTT. (c) Plasma insulin concentration during IVGTT. (d) Area under curve for insulin during IVGTT. Data represent mean ± SEM. **P<0.01 by Two-way ANOVA (a and c) and Student's t-test (b and d).

Supplementary Fig. 9

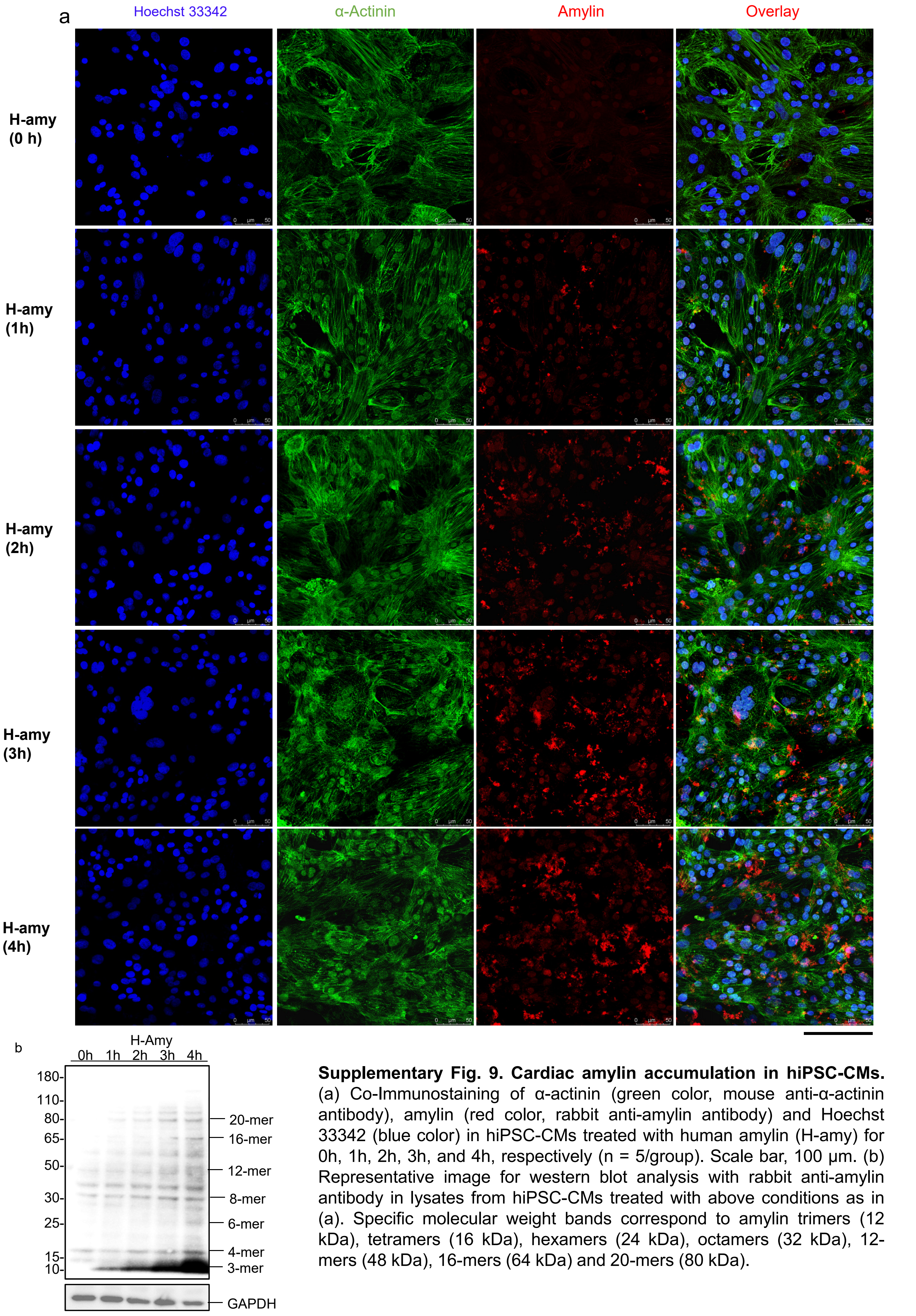

Supplementary Fig. 9. Cardiac amylin accumulation in hiPSC-CMs. (a) Co-Immunostaining of α-actinin (green color, mouse anti-α-actinin antibody), amylin (red color, rabbit anti-amylin antibody) and Hoechst 33342 (blue color) in hiPSC-CMs treated with human amylin (H-amy) for 0h, 1h, 2h, 3h, and 4h, respectively (n = 5/group). Scale bar, 100 μm. (b) Representative image for western blot analysis with rabbit anti-amylin antibody in lysates from hiPSC-CMs treated with above conditions as in (a). Specific molecular weight bands correspond to amylin trimers (12 kDa), tetramers (16 kDa), hexamers (24 kDa), octamers (32 kDa), 12-mers (48 kDa), 16-mers (64 kDa) and 20-mers (80 kDa).

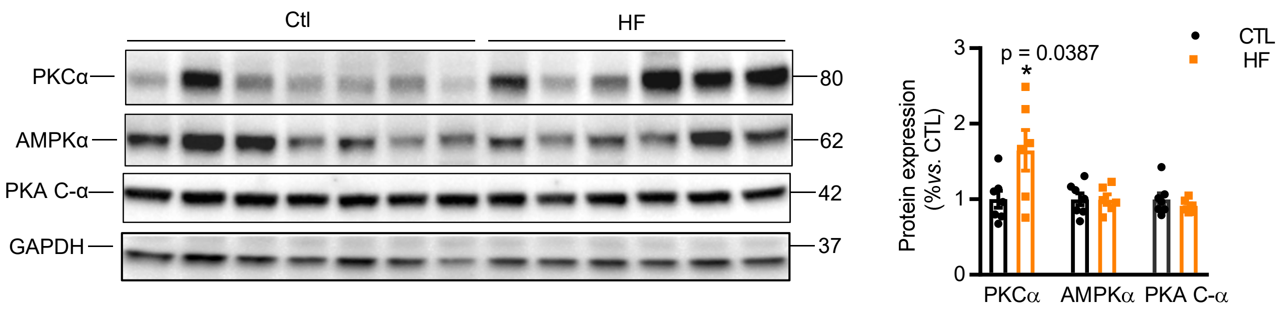

Supplementary Fig. 10. AMPKα, PKA C-α and PKCα expression levels in heart from NHPs with CTL and HF. Alterations in the protein expression of AMPKα, PKA C-α and PKCα in hearts from NHPs with CTL ($n = 7$) and HF ($n = 6$). Data represent mean ± SEM. *$P<0.05$ by Student's t-test.

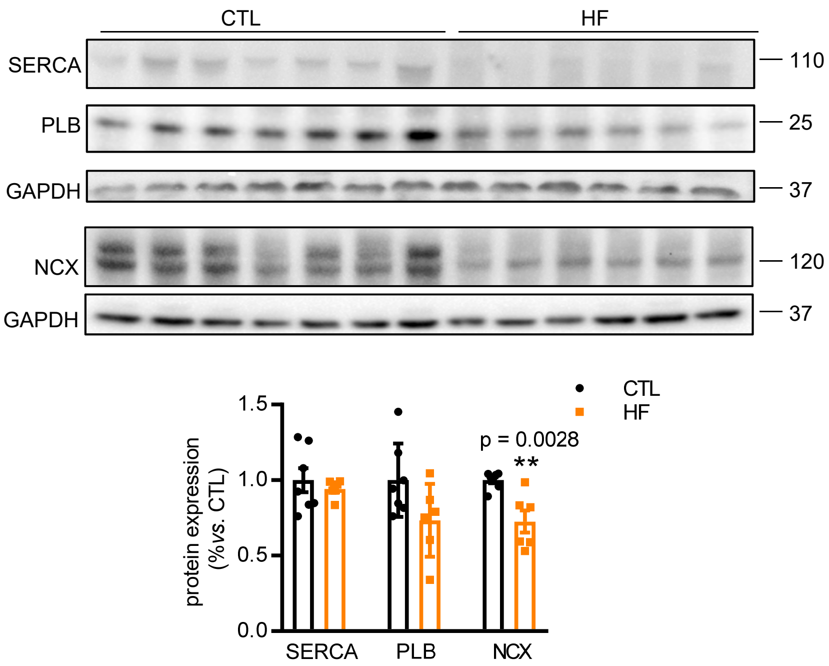

Supplementary Fig. 11. Altered Ca^{2+} cycling proteins in hearts from NHPs with CTL and HF. Alterations in the protein expression of sarcoplasmic reticulum Ca^{2+}-ATPase (SERCA), phospholamban (PLB) and Na^+/Ca^{2+} exchanger (NCX) in hearts from NHPs with CTL (n = 7) and HF (n = 6). Data represent mean ± SEM. **P<0.01 by Student's t-test.

Supplementary Fig. 12

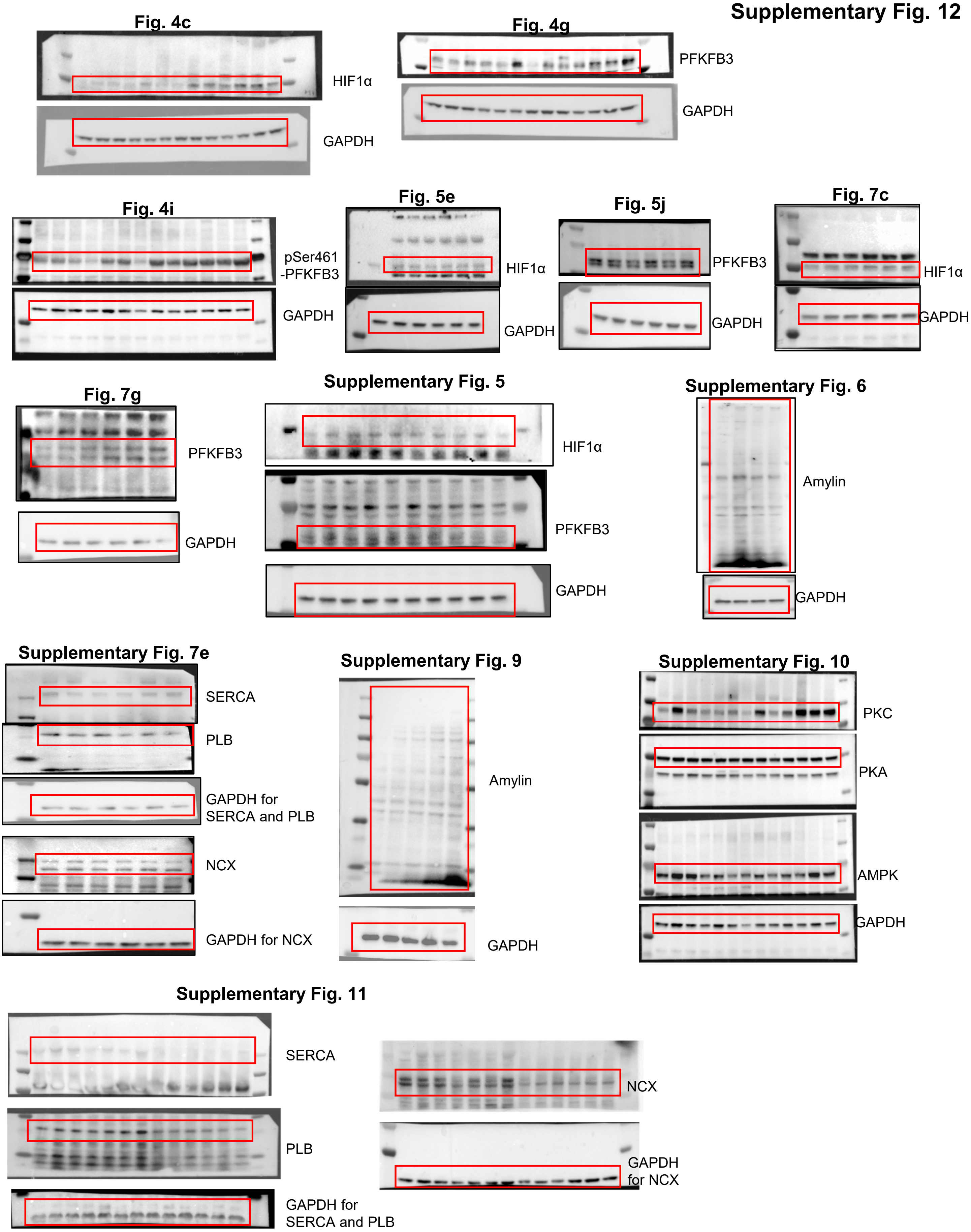

Supplementary Fig. 12. The full, uncropped blot images used in the manuscript.

Supplementary Table 1. Age, gender, body weight and BMI for all NHPs whose heart tissue was used in this study.

Group	Age (years)	Gender	Body weight (kg)	BMI (kg/m^2)
Control (CTL)	7	M	5.1	24.54
	9	M	5.5	26.49
	6	M	4.8	24.16
	17	F	3.0	17.23
	15	F	2.6	15.97
	16	M	5.7	28.99
	18	M	6.2	28.44
Heart failure (HF)	20	M	8.5	45.75
	14	M	10.6	57.17
	19	M	8.8	52.29
	22	F	3.4	19.27
	15	M	9.5	59.38
	17	M	9.2	49.76

Supplementary Table 2. Grading standards for major histological finding in hearts of NHPs used in this study.

Grade	0-<br>No apparent change	1+<br>minimal change	2+<br>moderate change	3+<br>marked change	4+<br>severe change	Note
**Arteriopathy**	no apparent change in different size artery	<10% increase in thickness of the tunica media of the vascular smooth muscles, with or without cell number change	10-30% increase in thickness of the tunica media of the vascular smooth muscles, with or without cell number change; 10-30% decrease in lumen	30 -50% increase in thickness of the tunica media of the vascular smooth muscles, with or without cell number change; 30-50% decrease in lumen	> 50% increase in thickness of the tunica media of the vascular smooth muscles, with or without cell number change; > 50% decrease in lumen	All sizes of arteries in heart were evaluated
**Degeneration/ necrosis**	no apparent change	perinuclear halo or vacuolation in cytoplasm, focal	perinuclear halo or vacuolation in cytoplasm, multifocal	vacuoles or deposits in cytoplasm and/or nuclear, along with loss of stratification, necrotic tissue with inflammatory response, multifocal to coalescing	vacuoles or deposits in cytoplasm and/or nuclear, along with loss of stratification, necrotic tissue with inflammatory response, coalescing to diffuse	Cytoplasmic area and myofibrils change
**Fibrosis**	no apparent change	fibrosis, focal	fibrosis, multifocal	fibrosis, multifocal to coalescing	fibrosis, coalescing to diffuse	Masson’s trichrome stain and Picro Sirius red stain to quantify fibrotic area
**Hypertrophy**	no apparent change	pleomorphism in shape with enlarged myofiber, focal	pleomorphism in shape with enlarged myofiber, multifocal	large and bizarre nuclei, often annular in shape or double and pyknotic, with enlarged myofiber (with or without deposition of degenerative substance), focal	large and bizarre nuclei, often annular in shape or double and pyknotic, with enlarged myofiber (with or without deposition of degenerative substance), multifocal	Evaluate both hypertrophy and nuclear change in cardiomyocyte
**Inflammatory cell infiltration**	no Inflammatory cell infiltration	inflammatory cell infiltrated, focal	inflammatory cell infiltrated, multifocal	inflammatory cell infiltrated, multifocal to coalescing	inflammatory cell infiltrated, coalescing to disuse	Infiltrated immune cell was marked by using IHC to quantify the subtypes

Supplementary Table 3. Primers information

Gene name	Forward primer sequence	Reverse primer sequence
GAPDH (Human)	GCACCGTCAAGGCTGAGAAC	AGGGATCTCGCTCCTGGAA
HIF1α (Human)	AGCTTGCTCATCAGTTGCCA	CCAGAAGTTTCCTCACACGC
PFKFB3 (Human)	AAACTGACGCCTGTCGCTTA	GATGCGAGGCTTTTTGGTGG
GAPDH (NHPs)	TTTTCTCTTGCATCGCCAGC	ATGACGAGCTTCCCGTTCTC
HIF1α (NHPs)	GGACTTGCCTTGCCTTCTCT	TTTTTCTTGTCGTTCGCGCC
PFKFB3 (NHPs)	TCCCAGAGCTTTCCGAGCG	GAAACGTGCTTTGGGCTAAGTT
GAPDH (Rat)	AGTGCCAGCCTCGTCTCATA	GATGGTGATGGGTTTCCCGT
HIF1α (Rat)	GCAACTGCCACCACTGATGA	GCTGTCCGACTGTGAGTACC
PFKFB3 (Rat)	CGCAATAGTGTCACCCCACT	TCCCTAGCAAAGGTTGTCCG
