# Supplementary material for: Amylin deposition activates HIF1α and 6-phosphofructo-2-kinase/fructose-2, 6-biphosphatase 3 (PFKFB3) signaling in failing hearts of non-human primates
Source: Commun Biol. 2021 Feb 12;4:188. doi: 10.1038/s42003-021-01676-3 (PMC7881154; doi:10.1038/s42003-021-01676-3)
Supplement: Supplementary file 3 — Description of Additional Supplementary Files [file 42003_2021_1676_MOESM3_ESM.pdf]

Description of Additional Supplementary Items

File Name: Supplementary Data 1

Description: Source data for the main and supplementary figures
