# Supplementary material for: Amylin deposition activates HIF1α and 6-phosphofructo-2-kinase/fructose-2, 6-biphosphatase 3 (PFKFB3) signaling in failing hearts of non-human primates
Source: Commun Biol. 2021 Feb 12;4:188. doi: 10.1038/s42003-021-01676-3 (PMC7881154; doi:10.1038/s42003-021-01676-3)
Supplement: Supplementary file 5 — Reporting Summary [file 42003_2021_1676_MOESM5_ESM.pdf]

nature research

Corresponding author(s): Liangbiao George Hu

Last updated by author(s): Dec 21, 2020

# Reporting Summary

Nature Research wishes to improve the reproducibility of the work that we publish. This form provides structure for consistency and transparency in reporting. For further information on Nature Research policies, see our Editorial Policies and the Editorial Policy Checklist.

## Statistics

For all statistical analyses, confirm that the following items are present in the figure legend, table legend, main text, or Methods section.

| n/a | Confirmed | |
|---|---|---|
| ☐ | ☒ | The exact sample size (*n*) for each experimental group/condition, given as a discrete number and unit of measurement |
| ☒ | ☐ | A statement on whether measurements were taken from distinct samples or whether the same sample was measured repeatedly |
| ☐ | ☒ | The statistical test(s) used AND whether they are one- or two-sided<br>*Only common tests should be described solely by name; describe more complex techniques in the Methods section.* |
| ☒ | ☐ | A description of all covariates tested |
| ☒ | ☐ | A description of any assumptions or corrections, such as tests of normality and adjustment for multiple comparisons |
| ☒ | ☐ | A full description of the statistical parameters including central tendency (e.g. means) or other basic estimates (e.g. regression coefficient) AND variation (e.g. standard deviation) or associated estimates of uncertainty (e.g. confidence intervals) |
| ☒ | ☐ | For null hypothesis testing, the test statistic (e.g. *F*, *t*, *r*) with confidence intervals, effect sizes, degrees of freedom and *P* value noted<br>*Give P values as exact values whenever suitable.* |
| ☒ | ☐ | For Bayesian analysis, information on the choice of priors and Markov chain Monte Carlo settings |
| ☒ | ☐ | For hierarchical and complex designs, identification of the appropriate level for tests and full reporting of outcomes |
| ☒ | ☐ | Estimates of effect sizes (e.g. Cohen's *d*, Pearson's *r*), indicating how they were calculated |

*Our web collection on statistics for biologists contains articles on many of the points above.*

## Software and code

Policy information about availability of computer code

| | |
|---|---|
| Data collection | FlexStation 3 reader (FLIPRTetra, Molecular Devices), Microplate reader (SpectraMax M5, Molecular Devices). Leica Aperio AT2 scanner (200x magnification; Leica Biosystems),confocal microscope (Leica TCS SP8, Germany),optical mapping system OMS-PCIE-2002 (Mapping Lab, UK),and BD FACSDiva software. |
| Data analysis | Halo software v3.0.311.217 (Indica Labs) with Area Quantification (v2.1.3.0; Indica Labs) and CytoNuclear (v2.0.5.0; Indica Labs) was used to analyzed all histochemistry data. GraphPad Prism 7.04 was used to analyze the statistic data. |

For manuscripts utilizing custom algorithms or software that are central to the research but not yet described in published literature, software must be made available to editors and reviewers. We strongly encourage code deposition in a community repository (e.g. GitHub). See the Nature Research guidelines for submitting code & software for further information.

## Data

Policy information about availability of data

All manuscripts must include a data availability statement. This statement should provide the following information, where applicable:

- Accession codes, unique identifiers, or web links for publicly available datasets
- A list of figures that have associated raw data
- A description of any restrictions on data availability

All data generated and analyzed during this study are included in this article and supplementary information files. Additional data and information about this study are available from the corresponding author upon request.

# Field-specific reporting

Please select the one below that is the best fit for your research. If you are not sure, read the appropriate sections before making your selection.

☒ Life sciences ☐ Behavioural & social sciences ☐ Ecological, evolutionary & environmental sciences

For a reference copy of the document with all sections, see nature.com/documents/nr-reporting-summary-flat.pdf

# Life sciences study design

All studies must disclose on these points even when the disclosure is negative.

| | |
|---|---|
| Sample size | No sample size calculation was performed. Sample size was determined to be adequate based on prior literature and the consistency of measurable differences between groups. |
| Data exclusions | N/A |
| Replication | The experiments using cell lines were replicated 3 times. |
| Randomization | N/A |
| Blinding | All analyses were conducted with blinding to the experimental condition. |

# Reporting for specific materials, systems and methods

We require information from authors about some types of materials, experimental systems and methods used in many studies. Here, indicate whether each material, system or method listed is relevant to your study. If you are not sure if a list item applies to your research, read the appropriate section before selecting a response.

## Materials & experimental systems

| n/a | Involved in the study | |
|---|---|---|
| ☐ | ☒ | Antibodies |
| ☐ | ☒ | Eukaryotic cell lines |
| ☒ | ☐ | Palaeontology and archaeology |
| ☐ | ☒ | Animals and other organisms |
| ☒ | ☐ | Human research participants |
| ☒ | ☐ | Clinical data |
| ☒ | ☐ | Dual use research of concern |

## Methods

| n/a | Involved in the study | |
|---|---|---|
| ☒ | ☐ | ChIP-seq |
| ☐ | ☒ | Flow cytometry |
| ☒ | ☐ | MRI-based neuroimaging |

## Antibodies

Antibodies used

anti-α-alpha actinin (sarcomeric) antibody (1:800, AF7811, Sigma-Aldrich),
anti- HIF1α antibody (1:200, ab16066, Abcam),
anti-HIF1α antibody (1:20, AF1935, R&D systems),
anti-PFKFB3 antibody (1:100, ab181861, Abcam),
anti-phospho461-PFKFB3 antibody (ab202291, Abcam),
rabbit anti-amylin antibody (1:400, T4157, Peninsula Laboratories, San Carlos, CA),
anti-amylin antibody (1:50, sc377530, Santa Cruz, Dallas, TX),
anti-SERCA (1:1000, MA3-919, Thermo Fisher, Waltham, MA),
anti-NCX (1:1000, MA3-926, Thermo Fisher, Waltham, MA),
anti-phospholamban (1:1000, PA5-82945, Thermo Fisher, Waltham, MA),
anti-GAPDH (1:10000, MA515738, Thermo Fisher, Waltham, MA)
anti-CD3 (CME324C, Biocare Medical),
anti-CD8 (ab178089, Abcam),
anti-CD45 (M0701, Dako),
anti-CD68 (CM033B, Biocare Medical)
anti-PKA C-α(4782S, Cell Signaling Technology)
anti-PKC α(2056S, Cell Signaling Technology)
anti-AMPKα (2532s, Cell Signaling Technology)

Validation

anti-α-alpha actinin (sarcomeric) antibody (1:800, AF7811, Sigma-Aldrich) was validated for detection of α-alpha actinin by its blocking peptide: α-actinin peptide (AT01, Cytoskeleton, Inc) by immunofluorescence and by knockdown its expression with small interference RNA:siActinin (siRNA ID: s968, 4392420, Thermo Fisher) by WB.
anti- HIF1α antibody (1:200, ab16066, Abcam) was validated for detection of HIF1α by its blocking peptide: HIF1α peptide (ab154478, Abcam) by immunofluorescence and by knockdown its expression with its small interference RNA:siHIF1α (siRNA ID:

s6539, 4390824, Thermo Fisher) by WB.,
anti-HIF1α antibody (1:20, AF1935, R&D systems) was validated for detection of HIF1α by its blocking peptide: HIF1α peptide (ab154478, Abcam) by immunofluorescence.
anti-PFKFB3 antibody (1:100, ab181861, Abcam) was validated for detection of PFKFB3 protein via transfection of PFKFB3 plasmid to HEK-293 cell line by WB and IHC.
anti-phospho461-PFKFB3 antibody (ab202291, Abcam) was validated for detection of hospho461-PFKFB3 protein via transfection of PFKFB3 S461A mutant plasmid to HEK-293 cell line by WB.
anti-amylin antibody (1:400, T4157, Peninsula Laboratories, San Carlos, CA) was validated for detection of amylin protein by its blocking peptide: amylin peptide (AS-60254-1, Anaspec) by immunofluorescence.
anti-amylin antibody (1:50, sc377530, Santa Cruz, Dallas, TX) was validated for detection of amylin protein via transfection of amylin plasmid to HEK-293 cell line by WB and IHC.
anti-SERCA (1:1000, MA3-919, Thermo Fisher, Waltham, MA) was validated for detection of SERCA by knock down its expression with its small interference RNA and tested in WB.
anti-NCX (1:1000, MA3-926, Thermo Fisher, Waltham, MA) was validated for detection of NCX via transfection of NCX plasmid to HEK-293 cell line by WB and immunofluorescence.
anti-phospholamban (1:1000, PA5-82945, Thermo Fisher, Waltham, MA) was validated for detection of PLB by Relative expression to ensure that the antibody binds to the PLB in IHC.
anti-GAPDH (1:10000, MA515738, Thermo Fisher, Waltham, MA) was validated for detection of GAPDH by WB.
anti-CD3 (CME324C, Biocare Medical), anti-CD8 (ab178089, Abcam), anti-CD45 (M0701, Dako), anti-CD68 (CM033B, Biocare Medical) were validated by their relative isotype control in IHC.
anti-PKA (1:1000, 4782S, Cell Signaling Technology), anti-PKC (1:1000, 056S, Cell Signaling Technology), anti-AMPK (1:1000, 2532s, Cell Signaling Technology) were validated by the vendor

## Eukaryotic cell lines

Policy information about cell lines

| | |
|---|---|
| Cell line source(s) | human induced pluripotent stem cell-derived cardiomyocytes (hiPSC-CMs) were bought from Help Stem Cell Innovation. |
| Authentication | The authentication procedures for this cell line was tested by vendor. |
| Mycoplasma contamination | The cell line were not tested for mycoplasma contamination |
| Commonly misidentified lines (See ICLAC register) | *Name any commonly misidentified cell lines used in the study and provide a rationale for their use.* |

## Animals and other organisms

Policy information about studies involving animals; ARRIVE guidelines recommended for reporting animal research

| | |
|---|---|
| Laboratory animals | Heart tissues and blood used from these animals cynomolgus monkey (Macaca fascicularis) age: 6-19, sex:male, female were purchased from Wuxi AppTec and Kunming Biomedical International; Fresh hearts from Sprague dawley rats: male, 2 month old were purchased from Wuxi AppTec. Fresh hearts from Amylin knock-out mice and WT mice were obtained from Shanghai Model Organisms. LTD. |
| Wild animals | N/A |
| Field-collected samples | N/A |
| Ethics oversight | All the protocols for animal experiments in this study were approved by the Experimental Animal Committee of Wuxi AppTec and Kunming Biomedical International and Shanghai Model Organisms. LTD. |

Note that full information on the approval of the study protocol must also be provided in the manuscript.

## Flow Cytometry

### Plots

Confirm that:

☒ The axis labels state the marker and fluorochrome used (e.g. CD4-FITC).

☒ The axis scales are clearly visible. Include numbers along axes only for bottom left plot of group (a 'group' is an analysis of identical markers).

☐ All plots are contour plots with outliers or pseudocolor plots.

☒ A numerical value for number of cells or percentage (with statistics) is provided.

### Methodology

| | |
|---|---|
| Sample preparation | After treatment and washing, the cells were stained using annexin V and propidium iodide (PI) for 15 min at room temperature and analysed using a BD LSRFortessa cell analyser BD LSRFortessa cell analyser |
| Instrument | BD LSRFortessa cell analyser |

| | |
|---|---|
| Software | BD FACSDiva software. |
| Cell population abundance | Cell populations were selected according to the size (forward-scatter) and complexity (side-scatter). Debris were excluded. 10,000 events per data point were collected for analysis. |
| Gating strategy | Cell populations were selected according to the size (forward-scatter) and complexity (side-scatter). Debris were excluded. 10,000 events per data point were collected for analysis. |

☐ Tick this box to confirm that a figure exemplifying the gating strategy is provided in the Supplementary Information.
